# Anti-Melanogenic Activities of *Heracleum moellendorffii* via ERK1/2-Mediated MITF Downregulation

**DOI:** 10.3390/ijms17111844

**Published:** 2016-11-04

**Authors:** Md Badrul Alam, Bum-Ju Seo, Peijun Zhao, Sang-Han Lee

**Affiliations:** Department of Food Science & Biotechnology, Kyungpook National University, Daegu 41566, Korea; mbalam@knu.ac.kr (M.B.A.); vicpie@knu.ac.kr (B.-J.S.); laputaily@hotmail.com (P.Z.)

**Keywords:** *Heracleum moellendorffii*, melanogenesis, tyrosinase, melanin, ERK, MITF

## Abstract

In this study, the anti-melanogenic effects of *Heracleum moellendorffii* Hance extract (HmHe) and the mechanisms through which it inhibits melanogenesis in melan-a cells were investigated. Mushroom tyrosinase (TYR) activity and melanin content as well as cellular tyrosinase activity were measured in the cells. mRNA and protein expression of microphthalmia-associated transcription factor (MITF), tyrosinase (TYR), TYR-related protein-1 (TYRP-1) and -2 were also examined. The results demonstrate that treatment with HmHe significantly inhibits mushroom tyrosinase activity. Furthermore, HmHe also markedly inhibits melanin production and intracellular tyrosinase activity. By suppressing the expression of TYR, TYRP-1, TYRP-2, and MITF, HmHe treatment antagonized melanin production in melan-a cells. Additionally, HmHe interfered with the phosphorylation of extracellular signal-regulated kinase (ERK) 1/2, with reversal of HmHe-induced melanogenesis inhibition after treatment with specific inhibitor U0126. In summary, HmHe can be said to stimulate ERK1/2 phosphorylation and subsequent degradation of MITF, resulting in suppression of melanogenic enzymes and melanin production, possibly due to the presence of polyphenolic compounds.

## 1. Introduction

Melanin, the major determining factor for skin color, also provides a defense system against the harmful effects of ultraviolet (UV)-induced skin damage. Abnormal melanin production manifests as several skin diseases such as albinism, leukoplakia, melasma, freckles, moles, and lentigo [1]. A series of complex oxidative and enzymatic reactions are involved in melanin biosynthesis; among them, tyrosinase is a key copper-containing enzyme that catalyzes the two rate-limiting steps of melanogenesis: hydroxylation of tyrosine to 3,4-dihydroxyphenylalanine (DOPA), and oxidation of DOPA to dopaquinone [2]. UV-induced melanin biosynthesis primarily results in the production of pheomelanin but also induces the continuous generation of reactive oxygen species (ROS), including hydrogen peroxide (H_2_O_2_), hydroxyl radicals, and superoxide radicals in melanocytes [3]. Additionally, oxidative intermediates such as reactive quinones, which are damaging to cellular proteins and DNA, are generated during melanogenesis [4]. H_2_O_2_ accumulation in normal melanocytes is directly proportional to melanin synthesis, allowing for the conclusion that the presence of reactive oxygen species (ROS) can lead to irregular melanogenesis, including the overproduction of melanin. Previous studies have informed that increased ROS/reactive nitrogen species (RNS) generation induced by ultraviolet radiation (UVR) correlates with an elevation in melanogenesis, possibly via the upregulation of tyrosinase activity, and protein and mRNA levels in melanocyte cells [5]. Formation of nitric oxide (NO) and oxidative damage were also found to mediate the promotion of melanin production in melanocytes by way of activation of the α-melanocyte stimulating hormone (α-MSH)/melanocortin 1 receptor (MC1R) or MITF signaling pathway [6]. Previous studies also revealed that UVR-induced ROS/RNS generation not only interfered with melanogenesis, but also contributed to melanocyte proliferation and transformation, which ultimately leads to melanogenesis [7]. Therefore, antioxidant defenses might play a beneficial role in mitigating the detrimental effect of excessive melanin production induced by oxidant formation. Researchers are developing various biological reductants and tyrosinase inhibitors such as kojic acid, sulfite, and arbutin to ameliorate hyperpigmentary disorders and pathological complexion discolorations [8,9]. However, whitening products containing potent tyrosinase inhibitors have severe side effects, including high cellular toxicity and low oxygen and water stability, thus limiting their application. Thus, due to their low toxicity and side effect profile, natural biomaterials are now under substantial consideration in the development of an effective and safe skin-depigmenting agent in the field of cosmetic and cosmeceutical industry.

Naturally occurring substances that demonstrate potent antioxidant potential have recently been shown to be beneficial for maintaining skin coloration. *Heracleum moellendorffii* Hance (Umbelliferae) is an edible wild herb; geographically, it grows widely in the fields and mountains of Korea, China, and Japan. The roots are used in traditional Oriental medicine as an anti-inflammatory for arthritis, backache, and fever. Extracts of its leaves have also been found to have various pharmacological activities, including detoxification and antioxidative activities [10,11]. Several studies on the chemical constituents of *Heracleum moellendorffii* Hance extract (HmHe) have isolated a number of flavonoids, monoterpenoids, sesquiterpenoids, coumarins, and polyacetylenic compounds [12,13]. The chemical composition of the essential oil derived from the roots and flowering aerial parts of HmHe have also been identified to have strong insecticidal activity [14]. However, thus there have been no studies on the possible skin-related application of the *Heracleum moellendorffii* Hance (HmH) leaves.

To develop novel and useful inner beauty-purpose nutraceuticals, the present study evaluated the effects of HmHe extracts in the context of melanin production in a melanocyte cell culture system, and for the first time, the underlying mechanism by which an extract of HmHe mitigates the production of melanin was determined: HmHe downregulates melanogenesis factors in melan-a cells by activating extracellular signal-regulated kinase (ERK)1/2 signaling pathways.

## 2. Results

### 2.1. Chemical Compounds of HmHe by HPLC

**Figure 1:**
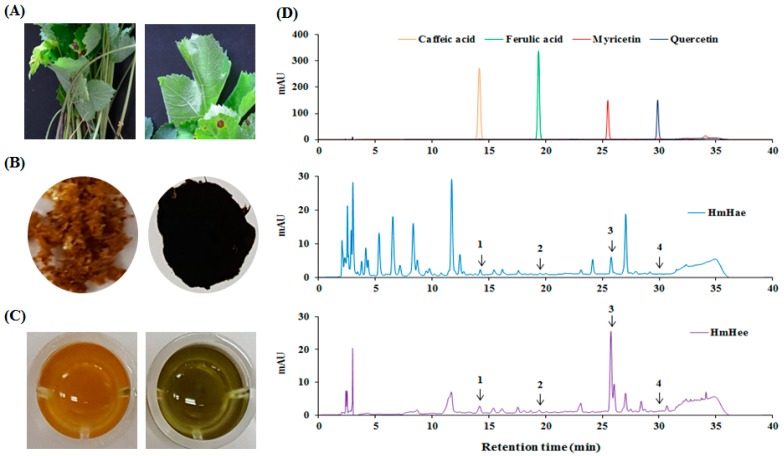
A classical feature of *Heracleum moellendorffii* Hance and HPLC chromatogram of the extracts monitored at 280 nm. A classical feature of *Heracleum moellendorffii* Hance leaf (**A**) and its powder (**B**) after fractionation is shown. The HmHae (**left** image of **C**) and HmHee (**right** image of **C**) solution were applied to a HPLC column and analyzed characteristic polyphenol compounds (**D**), as described in detail in Materials and Methods. **1**: caffeic acid, **2**: ferulic acid, **3**: myricetin, **4**: quercetin.

**Figure 2:**
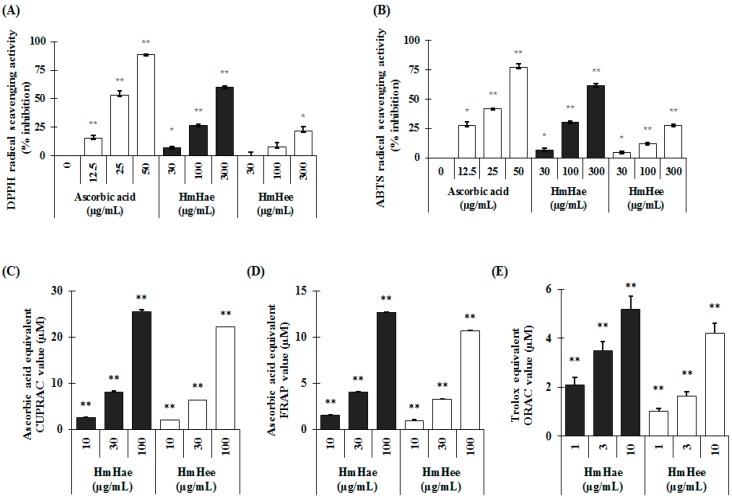
Antioxidant properties of *Heracleum moellendorffii* Hance by various in vitro antioxidant assays. DPPH radical scavenging activity (**A**); ABTS radical scavenging activity (**B**); CUPRAC activity (**C**); FRAP activity (**D**) and ORAC activity (**E**) were analyzed as described in detail in Materials and Methods. Each experiment was performed in triplicate and the data shown represent the mean ± SD. * *p* < 0.05, ** *p* < 0.01, student’s *t*-test.

Total polyphenol and flavonoid content were analyzed according to the Folin-Ciocalteu method [15] and aluminium chloride colorimetric assay [16]. In this study, using distilled water (DW) resulted in a higher extraction yield (10.22 ± 2.52) than using ethanol (2.36 ± 0.14). Similar results were also found in the case of total polyphenolic content, expressed as mg gallic acid equivalent per g dry extract (mg GAE/g), and flavonoid content, expressed as mg catechin equivalent per g dry extract (mg CE/g). *Heracleum moellendorffii* Hance aqueous extract (HmHae) has high polyphenolic content (280.50 ± 7.12 mg GAE/g), whereas the total polyphenolic content of *Heracleum moellendorffii* Hance ethanol extract (HmHee) was lower, at 223.14 ± 6.02 mg GAE/g. Surprisingly, HmHae also has high flavonoid content than HmHee, at 81.76 ± 2.23 mg CE/g and 54.07 ± 0.23 mg CE/g, respectively. HPLC analysis was performed to confirm the presence of polyphenol and flavonoids compounds in the extracts (Figure 1D). Table 1 shows the name of compounds that are confirmed by HPLC analysis (Figure 1D) and quantified with respect to the specific standard (data not shown). Antioxidants protect the cell from damage from oxidative stress. Antioxidant capacity may be defined as the scavenging of free radicals and reactive oxygen and nitrogen species by donating hydrogen or electrons. To investigate whether HmHe leaf extracts have antioxidant potential with respect to radical scavenging activities, 2,2-diphenyl-1-picrylhydrazyl- (DPPH) and 2,2′-azinobis-(3-ethylbenzothiazoline)-6-sulfonic acid (ABTS) -radical scavenging activities were examined. Both aqueous and ethanolic extracts of HmHe leaves significantly scavenged DPPH^•^, a stable organic nitrogen radical, as well as 2,2′-azinobis-(3-ethylbenzothiazoline)-6-sulfonic acid (ABTS^•+^), a mixed electron transfer and hydrogen atom transfer assay, in a dose-dependent manner (Figure 2A,B). Additionally, to confirm the electron-donating potentiality of HmHe, cupric-reducing antioxidant capacity (CUPRAC), ferric-reducing antioxidant power (FRAP) and oxygen radical absorbance capacity (ORAC) were assessed, and HmHae and HmHee were found to have a strong reducing capacity in a concentration-dependent manner (Figure 2C–E). Based on these observations, it is speculated that HmHe has a very strong capacity to scavenge various free radicals through hydrogen atom transfer and electron donation.

To further confirm whether anti-oxidant activity is associated with anti-melanogenic potential, we measured Pearson’s analysis by comparing the both activities. Interestingly, the results showed that antioxidant capacities of HmHae and HmHee ranked remarkable scores by exhibiting Pearson’s score as *p* = 0.998 and *p* = 0.995, respectively for anti-tyrosinase activity, and *p* = 0.979 and *p* = 0.931, respectively, for anti-melanogenic activity (data not shown).

### 2.2. Effects of HmHe on Mushroom Tyrosinase Activity, Melanin Production and Intracellular Tyrosinase Activity

**Figure 3:**
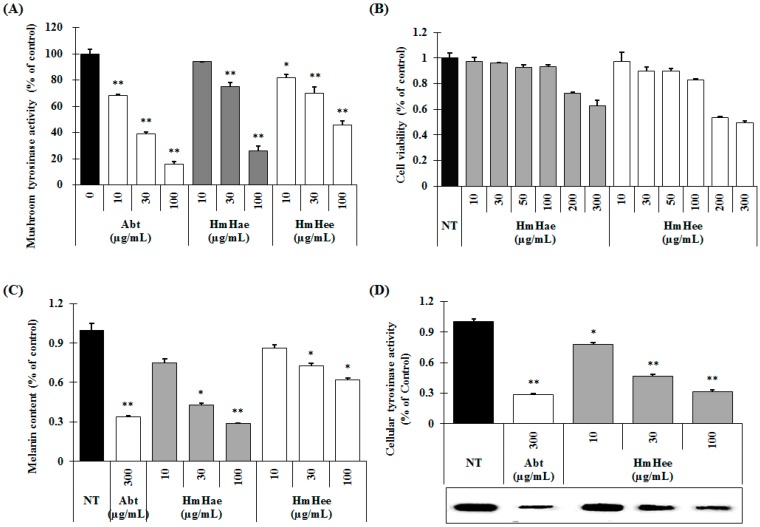
Inhibitory effects of HmHe leaf extracts on mushroom tyrosinase activity, melan-a cell viability, melanin content, and intracellular tyrosinase activity. (**A**) Different concentrations of the leaf extracts (10, 30, and 100 μg/mL) or arbutin were incubated with the same units of mushroom tyrosinase. Following incubation, the amount of dopachrome produced was determined at 490 nm spectrophotometrically; (**B**) Cells (1 × 10^5^ cells/mL) were seeded overnight, and the medium replaced with fresh medium containing the indicated concentrations of HmHae and HmHee or Arbutin. Cells were then cultured for 72 h and further incubated for 24 h after washing with PBS. Afterwards, an MTT solution was applied and the cells were further incubated for 1 h before cell viability was determined spectrometrically at 595 nm; (**C**,**D**) Cells (1 × 10^5^ cells/mL) were seeded overnight, and the medium replaced with fresh medium containing the indicated concentrations of HmHae and HmHee or Arbutin. Cells were then cultured for 72 h and further incubated for 24 h. After washing with PBS, the cells were lysed with 250 μL 1N NaOH and transferred to a 96-well plate. The melanin content was measured at 405 nm spectrophotometrically. To measure intracellular tyrosinase activity, protein levels were quantified and tyrosinase activity was determined by l-3,4-dihydroxyphenylalanine (l-DOPA) zymography. Each experiment was performed in triplicate and the data shown represent the mean ± SD. * *p* < 0.05, ** *p* < 0.01, student’s *t*-test. Abt: arbutin, NT: not treated.

Mushroom tyrosinase is widely used as the target enzyme when screening potential inhibitors of melanogenesis. To investigate whether HmHe leaves have anti-melanogenic activity, the effects of HmHe leaf extracts on mushroom tyrosinase enzyme activity were examined. Figure 3A shows that both HmHae and HmHee inhibit the mushroom tyrosinase activity in a dose-dependent manner. At a concentration of 100 µg/mL, HmHae inhibits mushroom tyrosinase activity by 73.96% ± 2.06% (Figure 3A, 7th column), whereas arbutin, a well-known tyrosinase inhibitor, showed 83.79% ± 2.01% (Figure 3A, 4th column). Hence, it can be concluded that HmHe leaves are considered a good source of tyrosinase inhibitor.

The melanin content and cell viability of melan-a cells were examined following exposure to HmHae and HmHee. Treatment with HmHae and HmHee at concentrations ranging from 10–100 µg/mL had no cytotoxic effect on melan-a cells (Figure 3B). Next, the melanin content of melan-a cells was examined, and was found to be significantly reduced after exposure to HmHae in a dose-dependent manner (Figure 3C, 3rd to 5th column). Furthermore, to examine the mechanism behind the inhibitory effects of HmHae on melanogenesis more precisely, intracellular tyrosinase activity in melan-a cells was measured by l-3,4-dihydroxyphenylalanine (l-DOPA) zymography. As shown in Figure 3D (3rd to 5th columns), HmHae (10–100 µg/mL) treatment significantly escalating the inhibition of tyrosinase in melan-a cells (Figure 3D; 3rd to 5th column). In cellular tyrosinase activity, HmHae exhibited a powerful anti-tyrosinase activity with an half maximal inhibitory concentration (IC_50_) value of 27.8 µg/mL; consistent with this, the mitigation of l-DOPA zymography band density by 50% was about 26.2 µg/mL in melan-a cells.

### 2.3. Effects of HmHe on Expression of Melanogenesis-Related Proteins

**Figure 4:**
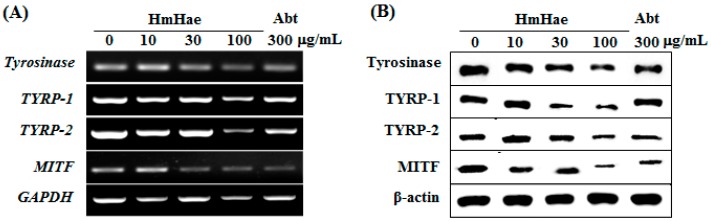
Effect of HmHae on the levels of melanogenesis-related mRNA and proteins in melan-a cells. (**A**) Cells (5 × 10^5^ cells/mL) were cultured for 24 h; the medium was replaced with fresh medium containing the indicated concentration of HmHae or arbutin for 24 h. mRNA was extracted using TRIzol, and expression was analyzed by reverse-transcription polymerase chain reactions; and (**B**) cells (1 × 10^5^ cells/mL) were cultured for 24 h; the medium was replaced with fresh medium containing the indicated concentrations of HmHae or arbutin for three days. Total cell lysate was extracted and assayed by Western blotting using antibodies against tyrosinase, TYRP-1, TYRP-2, and microphthalmia-associated transcription factor (MITF). Equal protein loading was confirmed using β-actin as a standard. Abt: arbutin.

To elucidate the mechanism of melanogenesis, including the expression of tyrosinase, TYRP-1, TYRP-2, and MITF, underlying the effect of HmHae, melan-a cells were treated with HmHae (10, 30, and 100 μg/mL) for five days. RT-PCR and Western blot analysis were conducted to analyze the resulting cell lysates. The effects of HmHae on the expression of melanogenic genes were investigated using RT-PCR. The results of mRNA expression analysis were consistent with the findings from protein analysis. mRNA levels of MITF and its downstream genes, tyrosinase, TYRP-1, and TYRP-2, were significantly abolished by HmHae (Figure 4A). As expected, compared with untreated control cells, HmHae treatment at 100 μg/mL significantly reduced tyrosinase, TYRP-1, TYRP-2, and MITF protein level in a dose-dependent manner (Figure 4B). These results indicate that HmHae may contribute to the inhibition of melanogenesis by regulating the expression of tyrosinase-related genes as well as MITF.

### 2.4. Effects of HmHe on ERK1/2 Signaling Pathways

**Figure 5:**
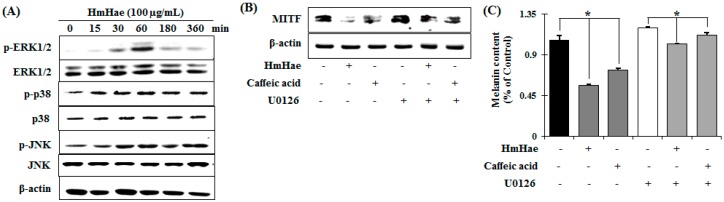
Effect of HmHae on mitogen-activated protein kinase (MAPK)-dependent signaling in melan-a cells. (**A**) Cells (5 × 10^5^ cells/mL) were cultured for 24 h, and the medium was replaced with fresh medium containing various concentrations of test compounds at the indicated times. Phosphorylation of c-Jun N-terminal kinases (JNK), extracellular signal–regulated kinases (ERK), and p38 MAPK was analyzed using phospho-specific JNK, ERK, and p38 MAPK antibodies by comparing non phospho-MAPKs. Equal protein loading was confirmed using β-actin antibodies; and (**B**) HmHae and caffeic acid were co-treated with a selective inhibitor (U0126) of ERK signaling molecules in melan-a cells. MITF was analyzed by Western blotting and (**C**) melanin content was also determined. Each determination was made in triplicate. Data represent the mean ± SD. * *p* < 0.05, versus the control group, Student’s *t*-test.

Melanin pigment formation and melanogenic gene expression is regulated by various signaling pathways including (primarily) MITF, as well as protein kinase A (PKA), cAMP response element-binding protein (CREB), mitogen-activated protein kinase (MAPKs), phosphatidylinositol-4,5-bisphosphate 3-kinase (PI3K), protein kinase B (AKT), and glycogen synthase kinase 3 β (GSK-3β) [17]. To elucidate the mechanism underlying the melanogenic effect of HmHae, melan-a cells were exposed to HmHae (100 µg/mL) for the indicated time points, and the protein extracts were then analyzed by Western blot analysis. As shown in Figure 5A, HmHae induced the phosphorylation of ERK1/2 at 60 min to 6 h after exposure. In contrast, JNK and p38 did not affect HmHae treatment in melan-a cells. These results suggest that the suppressive mechanism of HmHae is related to the activation of ERK signaling.

To investigate whether increased phosphorylation of ERK is associated with inhibition of MITF expression, pretreatment with U0126 (a selective inhibitor of the ERK pathway) was performed before HmHae treatment. Treatment with U0126 relieved HmHae-triggered inhibition of MITF expression (Figure 5B). To further scrutinize the effect of HmHae-induced ERK phosphorylation on melanin production, melanin contents were evaluated in the presence of U0126 in HmHae-treated cells. The level of melanin mitigated by HmHae was restored by U0126 treatment (Figure 5C). Thus, these results indicate that activation of the ERK pathway is involved in HmHae-induced reduction of melanogenesis.

## 3. Discussion

Since ancient times, Oriental countries like China, Korea and Japan have believed that a face with fair skin is the standard of beauty, and the admiration of women with young, bright, and fair skin has created a whitening cosmetics market. The color of human skin and hair is determined by a number of factors; among them, melanin biosynthesis (namely melanogenesis) is the most important factor. Melanogenesis is a multistage process involving melanin synthesis, melanin transport, and melanosome release. Although apt melanogenesis provides effective protection against harmful UV radiation, abnormal melanin production and accumulation lead to numerous dermatological disorders [18]. Researchers are developing various biological agents such as hydroquinone to ameliorate hyperpigmentary disorders and complexion discolorations. However, whitening products with potent tyrosinase inhibitor activity such as kojic acid, arbutin, and hydroquinone have severe side effects, including vitiligo, and skin peeling and redness, thus limiting their application [19,20]. Due to these side effects, natural ingredients with an inhibitory effect on melanin hyperpigmentation have been considered for the development of cosmetic agents in Oriental countries, with some medicinal chemists recently paying more attention to inhibitors of melanin production to prevent hyperpigmentary disorders such as melasma, freckles, and age spots [21]. In this study, HmHe extracts were found to significantly inhibit the mushroom tyrosinase activity. Furthermore, HmHae also mitigated cellular melanin production by inhibiting cellular tyrosinase activity. HmHae treatment significantly suppressed at a transcriptional and translational level the production of melanogenesis-related proteins in melan-a cells.

Tyrosinase plays a pivotal role in the melanin biosynthesis pathway. It converts l-tyrosine to l-DOPA and oxidizes l-DOPA to form dopachrome. Mushroom tyrosinase is widely used as the target enzyme in screening potential inhibitors of melanogenesis [22]. In the present study, HmHae and HmHee showed significant inhibitory effects on mushroom tyrosinase activity (Figure 3A). To elucidate the true inhibitory effect of HmHae and HLE on melanogenesis, melanin content and intracellular tyrosinase activity assays were performed at the same concentration range. As shown in Figure 3C, HmHae has a strong inhibitory effect on melanin production in melan-a cells than HmHee and arbutin. Furthermore, in accordance with the result of melanin content, HmHae also mitigated the intracellular tyrosinase activity in a dose-dependent manner (Figure 3D).

On the other hand, MITF is known to be involved in melanogenesis, and tyrosinase inhibitors control melanin production by suppressing the transcriptional as well as translational levels of melanin-related proteins such as tyrosinase, TYRP-1, TYRP-2, MITF, MC1R, ASIP (agouti signaling protein), and MGRN1 (mahogunin ring finger-1) [23]. Hence, in the present study, the effects of HmHae on the regulation of the transcriptional and translational expression of melanin related protein were investigated in melan-a cells. As expected, HmHae significantly mitigated the expression of both transcriptional and translational level of MITF, and its downstream enzymes such as tyrosinase, TYRP-1 and TYRP-2 (Figure 4). These results suggest that HmHae ameliorated melanogenesis by inhibiting the expression of tyrosinase, TYRP-1, and TYRP-2 through the inactivation of MITF in melan-a cells.

Previous studies have also shown that members of the MAP kinase family, ERK and JNK, play an important role in regulating melanogenesis [24]. Several studies have demonstrated that efficacious inhibitors of melanogenesis induce the phosphorylation of ERK and JNK, resulting in the phosphorylation of MITF at serine 73, which induces subsequent ubiquitin-dependent proteasomal degradation [25]. Thus, in the present study, to identify the specific mechanisms underlying the antimelanogenic activity of HmHae, ERK signaling molecules were analyzed by Western blot analysis in melan-a cells. HmHae at nontoxic concentrations effectively activated the phosphorylation of ERK in a time-dependent manner. Furthermore, co-treatment with HmHae, caffeic acid and ERK1/2 inhibitor significantly reversed HLA-induced decrease in MITF expression and, finally, melanin content (Figure 5).

Antioxidants protect cells from damage from oxidative stress. Antioxidant capacity may be defined as the scavenging of free radicals and reactive oxygen and nitrogen species by donating a hydrogen or electrons. Antioxidants are also well known to play a pivotal role in the antimelanogenesis of B16 cells [26]. Therefore, the antioxidative capacity of HmHae and HmHee on the basis of DPPH-, and ABTS-radical scavenging activity assays gives a good paradigm whether the fractions exhibit other inflammation-related diseases as well as symptoms. This can be compared by Pearson’s analysis between antioxidant and anti-melanogenic potentials (data not shown). The antioxidative capacity of HmHae was higher than that of HmHee. This result was in good agreement with the observed inhibitory effects of HmHae on the melanogenesis of melan-a cells, i.e., HmHae harboring a greater antioxidative capacity could further inhibit the melanogenesis of melan-a cells. Now, what are the components in HmHae responsible for both the antimelanogenic effects and the antioxidative capacity? It is noteworthy that *H. moellendorffii* is rich in flavonoids, monoterpenoids, sesquiterpenoids, and coumarins [12,13].

On the other hand, a recent study demonstrated that polyphenolic compounds with antioxidative potential showed inhibitory effects on the melanogenesis in B16 cells [27]. Therefore, in the present study, HPLC analysis was performed to identity the polyphenolic compounds, and the results are summarized in Table 1. Caffeic acid, ferulic acid, myricetin and quercetin were detected in leaves of *H. moellendorffii.* Interestingly, these polyphenols have been partially reported to have both antioxidative and anti-melanogenic effects in acerola [28]. As for the antimelanogenic effects of antioxidants, quercetin was shown to inhibit melanin biosynthesis in B16 cells via a decrease in the protein level of tyrosinase [29]. Plausibly, the inhibitory effects of HmHae on the melanogenesis of melan-a cells are attained through the downregulation of MITF and its upstream tyrosinase, TYRP-1 and TYRP-2, by the polyphenolic components.

Further studies on the antimelanogenic efficacy of the identified polyphenolic compounds in HmHae are rewarding in that the compounds in HmHe can be used for the development of functional food or novel whitening ingredients in the cosmetic, nutraceutical or inner beauty-purpose food industry.

## 4. Materials and Methods

### 4.1. Plant Materials and Extraction

*Heracleum moellendorffii* Hance was collected from a local supplier in Yeongyang, Korea, after cultivation in the spring of 2015. The plant material (Figure 1A) was taxonomically identified, and the voucher specimen (#2015-Hm) was maintained in our laboratory for future reference. The dried and coarsely powdered leaves were subjected to extraction thrice with 100% ethanol and water under reflux for 3 h, and the extracts (left and right images of Figure 1B) were filtered using a filter paper (No. 1 Whatman Schleicher Schuell, Keene, NH, USA). The supernatant was then collected and subjected to further lyophilization. Finally, the lyophilized samples were dissolved in deionized H_2_O to a concentration of 10 mg/mL (Figure 1C).

### 4.2. Drugs and Chemicals

Arbutin, 2,2-diphenyl-1-picrylhydrazyl (DPPH), l-tyrosine, l-DOPA (l-3,4-dihydroxy phenylalanine), ascorbic acid, Tween-20, thiazolyl blue tetrazolium bromide (MTT), 2,2′-azino-bis(3-ethylbenzthiazoline-6-sulfonic acid) (ABTS), *O*-tetradecanoyl phorbol-13-acetate (TPA), mushroom tyrosinase, 6-hydrolxy-2,5,7,8-tetramethylchroman-2-carboxylic acid (Trolox) were obtained from Sigma-Aldrich Co. (St. Louis, MO, USA). All other chemicals and reagents were high quality-grade and commercially obtainable. Antibodies purchased from Bioworld Technology (St. Louis Park, MN, USA) included anti-Tyr (Cat. No. BS6754), anti-TYRP-1 (Cat. No. sc-25543), anti-TYRP-2 (Cat. No. BS3320), anti-MITF (Cat. No. BS1550 for Western blotting), anti-phospho-JNK (Cat. No. 4668), anti-JNK (Cat. No. 9252), anti-phospho-p38 (Cat. No. 4511), anti-p38 (Cat. No. 9212), anti-p44/42MAPK (ERK1/2; Cat. No. 9102). Anti-phospho-p44/42MAPK (ERK1/2; Cat. No. 4376) and anti-MITF (Cat. No. 12590 for signaling) were obtained from Cell Signaling Technology (Beverly, MA, USA).

### 4.3. Chemical Compound Analysis by HPLC-DAD (High-Performance Liquid Chromatography with Diode-Array Detection)

To identify and quantify major polyphenol compounds in HmHe by HPLC, an Agilent 1200 chromatographic system (Agilent Technologies, Santa Clara, CA, USA) with UV-Vis diode array detector and ChemStation software (version, Palo Alto, CA, USA) was used. The samples were filtered through a 0.45 µm Nylon filter (E0034, Análisis Vínicos, Tomelloso, Spain) and low molecular weight polyphenolic compounds were analyzed according to a method described by Ohgidani et al. [30]. The Zorbax C18 column (150 × 4.6 mm, 5 µm particle size) (Agilent Technologies) maintained at 30 °C as well as DW-trifluoroacetic acid (90:10 *v*/*v*) and methanol-trifluoroacetic acid (80:20 *v*/*v*) were used as solvents A and B, respectively, for the separation of the polyphenolic compounds. The elution profile was as follows: 0–20 min, 80% of A; 20–25 min, 40% of A; 25–30 min, 0% of A; and 30–40 min, 20% of A. The flow rate was 0.8 mL/min, and the injection volume was 10 µL. The wavelengths of detection were 280 and 340 nm, respectively. Phenolic and flavonoid compounds were identified by their retention time. Polyphenolic compounds were identified by comparison of the retention times with those of available pure standards and quantification was carried out by using external calibration method with comparing the areas of standards of caffeic acid, ferulic acid, myricetin and quercetin.

### 4.4. Cell Culture and Cell Viability Assay

Melan-a, the melanocyte cell line, was obtained from Dorothy C. Bennett (St George’s, University of London, London, UK). The melan-a cells were cultivated in Roswell Park Memorial Institute (RPMI) 1640 medium supplemented with 10% fetal bovine serum (FBS, Hyclone, Utah, UT, USA), streptomycin-penicillin (100 µg/mL each), and 200 nM TPA at 37 °C in an incubator under a 5% CO_2_ condition. Cells were passaged every 3 days until a maximal passage number of 40 was reached. Confluent cell monolayers were harvested using a mixture of 0.05% trypsin and 0.53 mM ethylenediaminetetraacetic acid (Gibco BRL, Grand Island, NY, USA), and the cell viability was measured by a tetrazolium dye colorimetric test (MTT) kit. Melan-a cells were first cultured in a 96-well plate (1 × 10^5^ cells/well) for 24 h, washed twice using PBS, and pretreated with various concentrations of samples. After 24 h of incubation, the MTT reagent was added to each well and the plate was incubated at 37 °C for 1 h. The media was removed and the plate was washed twice with PBS (pH 7.4). The intracellular insoluble formazan was dissolved in 100% DMSO for measuring at 595 nm using a microplate reader (VICTOR3, PerkinElmer, Turku, Finland) and the percentage viability was calculated [31].

### 4.5. Measurement of Mushroom Tyrosinase Activity

Tyrosinase activity was measured as previously described [32]. Briefly, the reaction mixture contains 0.1 M phosphate buffer (pH 6.5) (100 µL), 1 mM l-tyrosine (50 µL) and mushroom tyrosinase (Enzyme Commission (EC) No. 1.14.18.1; 200 units/mL in phosphate buffer, pH 6.5) with or without various concentrations of samples (HmHae and HmHee) in a 96-well microplate (SPL, Pocheon, Korea). After taking the initial absorbance at 490 nm, the reaction mixture was incubated at 37 °C for 30 min, aging while taking the absorbance at the same wavelength using a microplate reader (VICTOR3). Tyrosinase inhibitory activity was then estimated using the following equation:

Inhibition activity (%) = [(A − B) − (C − D)]/(A − B) × 100,(1)

where A is the final absorbance of the control reaction (no HmHae or HmHee present); B is the initial absorbance of control; C is the final absorbance of the reaction in the presence of sample; and D is the initial absorbance of reaction.

### 4.6. Melanogenesis Inhibitory Assay in Melan-a Cells

Cells (1 × 10^5^ cells/mL) were seeded into a 24-well plate (BD Falcon, Bedford, MA, USA) and allowed to attach overnight. The medium was changed to a fresh medium containing various concentrations of samples and cultured further for 72 h. Cells were washed by PBS two times, and lysed with 1 N NaOH and transferred to a 96-well plate for measuring the melanin content at 405 nm using a microplate reader (VICTOR3). Arbutin was used as a positive control [33]. Then, the following equation was used to calculate the inhibition of melanogenesis:

Melanogenesis inhibition (%) = (A − B)/A × 100,(2)

where A is the absorbance of cells treated with sample or arbutin; and B is the absorbance of control.

### 4.7. Analysis of Intracellular Tyrosinase Activity by Zymography

Tyrosinase zymography was performed as described previously [34]. Cells (1 × 10^5^ cells/mL) were cultured with or without test samples (HmHae) for 72 h, washed with PBS twice, and harvested with Radioimmunoprecipitation assay (RIPA) cell lysis buffer supplemented with protease and phosphatase inhibitors. A Bicinchoninic acid (BCA) protein assay kit (Pierce Biotechnology, Rockford, IL, USA) was used to measure the concentration of protein. An equal amount (50 µg protein) of each sample was separated by 10% sodium dodecyl sulfate-polyacrylamide gel electrophoresis (SDS-PAGE). Thereafter, the gels were incubated in 0.1 M sodium phosphate buffer for 30 min with mild shaking followed by staining with 20 mM l-DOPA in 0.1 M sodium phosphate buffer at 37 °C for 1 h. Intracellular tyrosinase, separated by SDS-PAGE according to its molecular weight can be detected as a dark-colored dopaquinone following incubation with l-DOPA solution [35].

### 4.8. Analysis of mRNA Expressions

Total RNA was extracted from melan-a cells using TRIzol (Ambion, Austin, TX, USA), according to the manufacturer’s instructions [36]. To prepare a cDNA pool from each RNA, total RNA (2 μg) was transcribed using an RT- & GO Mastermix (MP Biomedicals, Seoul, Korea), and the product was used as the PCR template. Reverse transcription PCR (RT-PCR) was performed using PCR Thermal Cycler Dice TP600 (TAKARA Bio Inc., Otsu, Japan), and the following primer sequences were used: mouse tyrosinase (forward, 5′-CCC AGA AGC CAA TGC ACC TA-3′, reverse, 5′-ATA ACA GCT CCC ACC AGT GC-3′); mouse TYRP-1 (forward, 5′-GCT GCA GGA GCC TTC TTT CT-3′, reverse, 5′-AGA CGC TGC ACT GCT GGT C-3′); mouse TYRP-2 (forward, 5′-GGA TGA CCG TGA GCA ATG GC-3′, reverse, 5′-CGG TTG TGA CCA ATG GGT GC-3′); mouse MITF (forward, 5′-CAG GCT AGA GCG CAT GGA CT-3′, reverse, 5′-CTC CGT TTC TTC TGC GCT CA-3′); mouse glyceraldehyde-3-phosphate Dehydrogenase (GAPDH) as an internal control (forward, 5′-GC GAG ACC CCA CTA ACA TCA-3′, reverse, 5′-GAG TTG GGA TAG GGC CTC TCT T-3′). Each PCR product was analyzed on an agarose gel electrophoresis in Tris/Borate/EDTA (TBE) buffer at 100 V for 30 min. Then, each PCR product was stained by ethidium bromide, and the intensity of samples was normalized to that of GAPDH.

### 4.9. Preparation of Cell Lysates and Western Blotting

Melan-a cell lysates were prepared using a standard protocol, mixed with 5X SDS-PAGE (3M Science, Seoul, Korea) sample buffer and denatured at 100 °C for 5 min. An equal amount (20 µg protein) of each sample was separated by 10% SDS-PAGE gel electrophoresis, followed by electrotransfer to nitrocellulose membranes (Whatman, Dassel, Germany). The membranes were then incubated overnight with 5% skim milk (for anti-Tyr, anti-TYRP-1, anti-TYRP-2, anti-MITF) or 5% bovine serum albumin (BSA) for anti-phospho-JNK, anti-JNK, anti-phospho-p38, anti-p38, and anti-p44/42MAPK (ERK1/2), and anti-phospho-p44/42MAPK (ERK1/2), detection. anti-Tyr, anti-TYRP-1, anti-TYRP-2, anti-MITF (Bioworld Technology, St. Louis Park, MN, USA) for Western blotting, and anti-phospho-JNK, anti-JNK, anti-phospho-p38, anti-p38, and Anti-p44/42MAPK (ERK1/2), and anti-phospho-p44/42MAPK (ERK1/2), and anti-MITF (Cell Signaling Technology, Beverly, MA, USA) for signaling studies were utilized as 1st antibodies. Anti-goat IgG-horse radish peroxidase (HRP) and anti-mouse IgG-HRP were purchased from Santa Cruz and used as 2nd antibodies. The reaction was proceeded using an ECL system (Perkin Elmer).

### 4.10. In Vitro Antioxidant Assays

A 2,2-Diphenyl-1-picrylhydrazyl (DPPH) radical-scavenging assay was used for the evaluation of the free radical scavenging activity of the aqueous extract (HmHae), and an ethanolic extract (HmHee) of HmHe was conducted following the protocol described by Nanjo et al. [37], with minor modification. Briefly, a 198 μL of a 0.2 mM solution of DPPH in 50% ethanol was added to 2 μL of various concentrations of the sample. The mixture was allowed to stand at 25 °C for 10 min and the absorbance was measured at 517 nm in a multilevel counter (VICTOR3). Ascorbic acid was used as a standard antioxidant. The percent inhibition ability was calculated using the following equation:

Radical-scavenging activity (% inhibition) = [(Abs_control_ − Abs_sample_)/Abs_control_] × 100, (3)

where Abs_control_ is absorbance of control; and Abs_sample_ is the absorbance of the sample. All samples were analyzed in triplicate.

The method of Noguchi et al. [38] was adopted for the ABTS assay with slight modification. Varying concentrations of the samples (2 μL) were allowed to react with 198 μL of the ABTS^•+^ solution, and their respective absorbances were determined at 734 nm. Ascorbic acid was tested as the standard antioxidant. The percent inhibition ability was calculated using Equation (1).

To measure reducing power, the ferric reducing antioxidant power (FRAP) assay was performed as described previously [39] with a slight modification. Ascorbic acid was also used as the standard antioxidant, and the ascorbic acid equivalent FRAP value (µM) was calculated from its standard curve.

The cupric-reducing antioxidant capacity (CUPRAC) of HmHae and HmHee was determined according to the method by Apak et al. [40], with slight modifications. The ascorbic acid equivalent CUPRAC value (µM) was calculated from the standard curve of ascorbic acid.

The oxygen radical absorbance capacity (ORAC) assay was performed according to a previous report [39] in which trolox, a water-soluble analogue of vitamin E, was used as the positive control. After addition of 20 mM of 2,2′-Azobis (2-amidinopropane) dihydrochloride (AAPH), the analyzer was programmed to record the fluorescence of 200 nM fluorescein every minute, with a 480 nm excitation and a 520 nm emission wavelength. The results were calculated using the differences in the areas under the fluorescence decay curves between the blank sample and experimental sample, expressed as area under the curve (Net AUC) values.

### 4.11. Statistical Analysis

All data are shown as the mean ± SD. Data were analyzed using one-way ANOVA. Differences were considered significant if *p* < 0.05. All analyses were performed using SPSS for Windows, version 10.07 (SPSS, Chicago, IL, USA).

## Figures and Tables

**Table 1:** Quantified HPLC analysis of polyphenolic compounds from HmHae and HmHee.

Samples	Standards	Contents (mg/L)		
		280 nm	340 nm	Average ^1^
HmHae ^2^	Caffeic acid	13.16	12.41	12.80
	Ferulic acid	3.03	1.16	2.01
	Myricetin	103.95	96.12	100.04
	Quercetin	0.81	0.44	0.63
HmHee ^3^	Caffeic acid	25.98	21.90	23.9
	Ferulic acid	7.55	3.45	5.50
	Myricetin	446.19	408.84	427.52
	Quercetin	1.23	1.36	1.30

Results are determined between two independent experiments. ^1^ Sum of HPLC analysis monitored at [(280 nm and 340 nm)/2]; ^2^ *Heracleum moellendorffii* Hance leaf aqueous extract; ^3^ *Heracleum moellendorffii* Hance leaf ethanol extract.
